# Study on the Change of Dielectric Properties and Chemical-Mechanism during Coal Low-Temperature Oxidation

**DOI:** 10.1038/s41598-020-61584-0

**Published:** 2020-03-13

**Authors:** Hongqing Zhu, Haoran Wang, Wei Wang, Jiuli Liu

**Affiliations:** School of Emergency Management and Safety Engineering, China University of Mining and Technology (Beijing), Beijing, 100083 China

**Keywords:** Geochemistry, Geophysics

## Abstract

The study aims to investigate the variation of the coal dielectric properties during coal low-temperature oxidation and the effect of oxidation product on the coal dielectric properties. Four different types of coal were prepared under low-temperature oxidation condition, and the coal dielectric properties were measured with an impedance analyzer at frequencies ranging between 20 Hz and 30 MHz. The oxygen-containing functional groups in oxidized coal samples were semi-quantitatively evaluated using Fourier transform infrared spectroscopy. Low-temperature oxidation stage of coal spontaneous combustion could be predicted according to the change of coal dielectric properties in the process of temperature rise. It was found that the dielectric constant of coal with high water content decreased exponentially with temperature. For coal sample with low water content, the dielectric properties changed piecewise linearly with temperature. Coal dehydration was considered to be the reason for the decrease of the dielectric constant from 30 °C to 120 °C. The increase of the relative content of oxygen-containing functional groups, especially carbonyl compounds, could be the reason for the rise of the coal dielectric constant from *T*_*2*_ to *T*_*3*_.

## Introduction

Coal spontaneous combustion is one of the major disasters in collieries, and it had occurred in countries all over the world. Researchers reported that many of the coal spontaneous combustion areas were concealed^1,2^. Accurate detection of the high-temperature zone in the early low-temperature oxidation stage is a major interest within the coal spontaneous combustion prevention and control field. Electromagnetic detecting technology is a conventional coal fire detection technology. Some researchers had made progress in locating the coal fire area using magnetotelluric field monitoring equipment. However, this method is greatly affected by the content of magnetic mineral components in the coal seam, especially pyrite^3^. Some other researchers^4,5^ had tried to locate coal fire by ground-penetrating radar technique based on voids and clefts induced by coal fires. Magnetic mineral components do not restrict this method, but it may be affected by formation fractures. The limitations on electromagnetic detection of coal fire may be reduced if we can find the mapping relationship between coal dielectric constant and temperature in low-temperature oxidation stage. The prediction time may be advanced as well.

It is believed that the dielectric properties of coal are the basis for studying the propagation of electromagnetic waves in the coal seam. The dielectric properties are also the critical parameters for the development of various geological exploration instruments (e.g., ground-penetrating radar, transient electromagnetic (TEM) instruments, etc.)^6–10^. Therefore, it is of great theoretical and practical value to study the coal dielectric properties under low-temperature oxidation condition for the detection of concealed coal low-temperature oxidation areas. Many researchers had reported the dielectric properties of coal pyrolytic at high temperatures in the microwave band. They believed that the dielectric properties of pyrolysis coal were related to different composition and structure. Peng *et al*. and Wang *et al*. found that the dielectric properties of coal remain relatively constant below 500 °C, but the relative dielectric constant and loss factor increased sharply with the increase of temperature after 500 °C due to the release of volatiles^11,12^. Xu and co-workers found that the complex dielectric constant and the tangent of loss angle increase with the increment of temperature as a result of the ordered crystallite structure of coal^13,14^. S. Marland *et al*. found that due to moisture removal, substantial reductions in coal dielectric constant and loss factor values occur between 80 and 180 °C and the dielectric properties of coal are dependent on coal rank^15^. Xu *et al*. found that the dielectric constant of coal decreased with increasing temperature from 25 ° C to 120 ° C, and coal dielectric constant decreased first and then increased with the increment of coal rank^16^. Current studies have not dealt with the dielectric properties and corresponding chemical mechanism of coal at the low-temperature oxidation stage. However, this stage is believed to be the most critical stage for preventing and controlling spontaneous coal combustion^17–20^. Consequently, this paper attempts to show the dielectric property variation law of low-temperature oxidation coal and verify the component and structure differences among low-temperature oxidation coals at different temperatures and the resulting potential effects on the dielectric properties. A low-frequency electromagnetic waveband was used in this study considering the coal spontaneous combustion concealment nature.

This study aims to develop an understanding of the low-temperature oxidation effects on coal dielectric properties by investigating the relationship between low-temperature oxidation coal composition and structure and the resulting dielectric properties. The coal dielectric properties were measured with an E4990A impedance analyzer and the 16451B test fixture manufactured by Keysight in the frequency band of 20 Hz to 30 MHz. The relationship between the dielectric properties and component and structure of coal samples was measured and analyzed with a Bruker Tensor 27 Fourier transform infrared (FTIR) spectrometer. Moreover, the coal component and structure effect on the coal dielectric properties during the low-temperature oxidation process was elucidated.

## Experimental

### Preparation of coal samples

Coals were selected from four coal mines in China: lignite from Changji (CJ) in Xinjiang Region, coking coal from Wenshui (WS) in Shanxi Province, fat coal from Tangshan (TS) in Hebei Province, and meager-lean coal from Changzhi (CZ) in Shanxi Province. The coals were crushed below 200 mesh in an airtight crusher and placed in a brown wide-mouth bottle for later usage. The sampling strictly followed the Chinese national standards (GB/T 482-2008). Proximate analysis of the raw coal is summarized in Table 1.

**Table 1:** Proximate analyses of the coal samples.

Coal sample	Proximate analysis, wt%, dry basis			
	Moisture	Ash	Volatile	Fixed carbon
CJ coal	19.51	5.72	26.13	48.64
WS coal	0.89	13.97	21.97	63.17
TS coal	0.55	10.00	22.54	66.92
CZ coal	0.79	10.84	12.68	75.69

**Figure 1:**
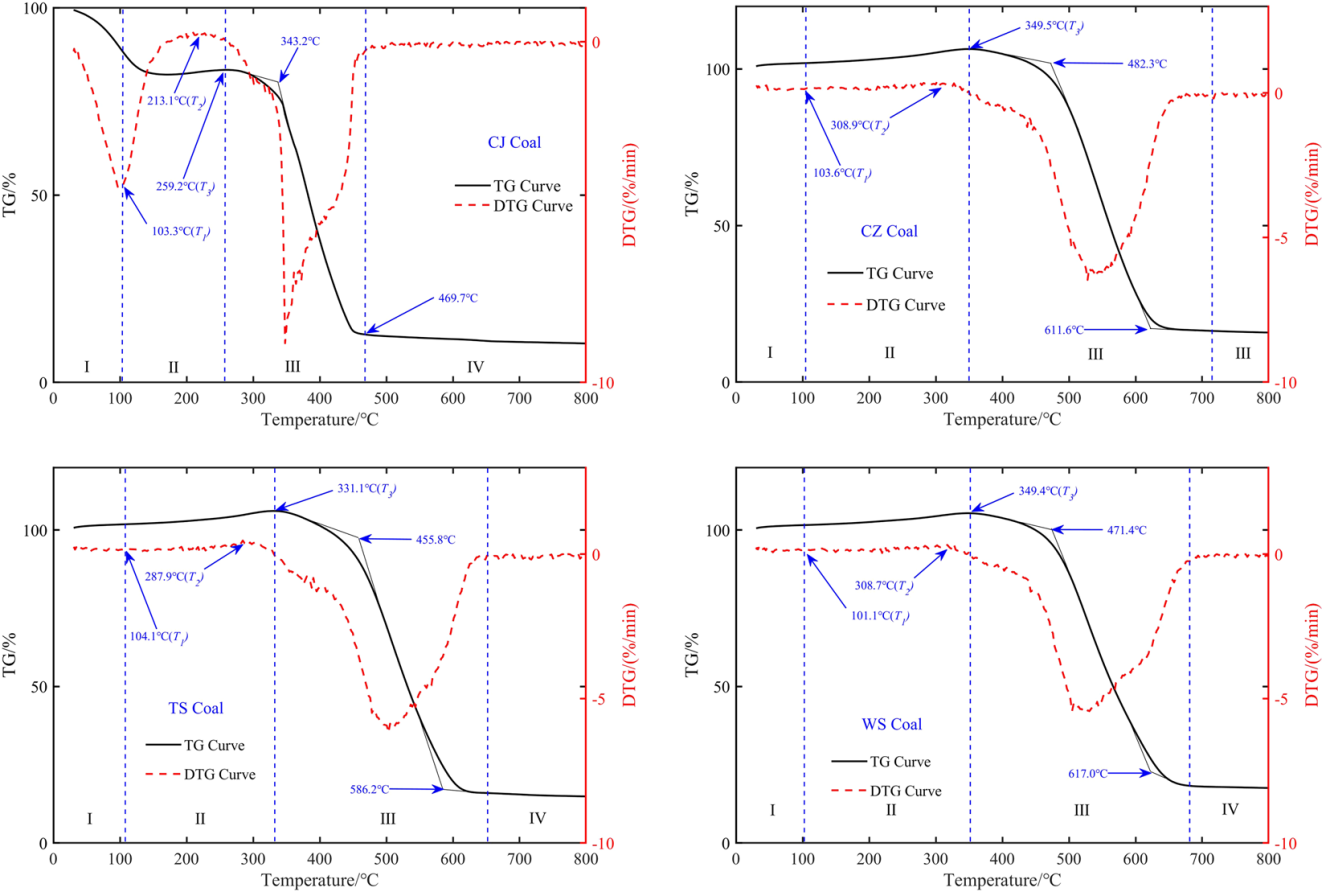
Characteristic temperatures of oxidized coal. I: Dehydration and gas desorption stage, II: Oxidation and weight increase stage, III: Combustion stage, IV: Burnout stage.

Previous research results had shown that coal with different ranks exhibited a series of similar temperature-dependent oxidation stages and characteristic temperatures under air atmosphere^21–25^. TG/DTG curves and characteristic temperatures of four kinds of coal samples at 10 K/min heating rate in the air atmosphere are shown in Fig. 1. It can be seen that the weight of the coal sample first decreases and then increases during 30 °C to *T*_*3*_. *T*_*1*_ is the minimum point on the DTG curve at low-temperature oxidation stage, which relates to maximum weight loss rate. As the moisture content of CZ, WS and TS coal samples is small, *T*_*1*_ is not as evident as CJ. *T*_*2*_ is the maximum point on the DTG curve at oxygen absorption stage, which associates with the maximum weight gain rate point. *T*_*3*_ is the maximum mass point on the TG curve at oxygen absorption stage. Some other temperatures were inserted in to reduce the test temperature intervals. The test temperatures were determined based on thermogravimetric experiments, as summarized in Table 2.

**Table 2:** Processing temperatures for coal samples under oxygen condition.

Coals	*T* (°C)								
CJ	30	70	103 (*T*_*1*_)	120	150	182	213 (*T*_*2*_)	230	260 (*T*_*3*_)
WS	30	70	103 (*T*_*1*_)	120	165	225	288	309 (*T*_*2*_)	350 (*T*_*3*_)
TS	30	70	103 (*T*_*1*_)	120	165	213	275	288 (*T*_*2*_)	330 (*T*_*3*_)
CZ	30	70	103 (*T*_*1*_)	120	165	225	288	309 (*T*_*2*_)	350 (*T*_*3*_)

The horizontal tube reactor preheated at a heating rate of 10 °C/min to characteristic temperatures. Following this, about 50 g coal powder was uniformly spread in a crucible and then pushed into the center of the heated tube. After been held at each characteristic temperature under air atmosphere for one hour, the coal power was naturally cooled to room temperature and then ground for later tests. Finally, 0.5 g coal powder and 1 g polyethylene was added to the mold and pressed into a 30-mm-diameter disc sample. The process was conducted under an air atmosphere to simulate coal spontaneous combustion.

### Measurement of dielectric properties

**Figure 2:**
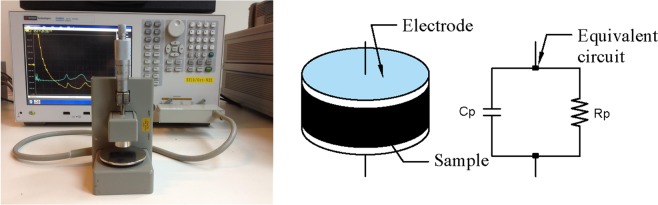
Keysight E4990A impedance analyzer and the equivalent circuit model.

The dielectric constant is described by Eq. (1) with two parts:

1 $$\varepsilon =\varepsilon {\prime} -j\varepsilon {\prime\prime} $$

where $\varepsilon {\prime}$, the real part of the complex dielectric constant $\varepsilon$ (F/m), and $\varepsilon {\prime\prime}$, the imaginary part of the dielectric constant (F/m), are related to polarisation and energy loss, respectively. The dielectric constant establishes the relationship between electric flux density D and electric field E. It is necessary for the macroscopic application of Maxwell-equations. The magnitudes of the measured real and imaginary dielectric constants are usually small. Thus they are generally re-scaled by dividing by the free space dielectric constant. The resulting value refers to the relative real dielectric constant ${\varepsilon {\prime} }_{{\rm{r}}}$ and relative imaginary dielectric constant ${\varepsilon {\prime\prime} }_{{\rm{r}}}$. The dielectric property of coal samples was assessed in the frequency range from 20 Hz to 30 MHz with an E4990A impedance analyser manufactured by Keysight. The parallel equivalent circuit was employed since the coal impedance value was large. The measurement instrument and the corresponding measurement principle are shown in Fig. 2.

The real part and imaginary part of the relative dielectric constant were calculated using Eqs. (2–4). Where *R*_*p*_ was the sample equivalent resistance, *t* was the sample thickness, ${\varepsilon }_{0}$ was the dielectric constant of a vacuum, and *d* was the diameter of the measurement electrode, which was 5 × 10^−3^ m.

2 $${\varepsilon }_{r}=({C}_{P}/{C}_{0}-jG/\omega {C}_{0})$$

3 $${\varepsilon {\prime} }_{r}=4t{C}_{P}/\pi {d}^{2}{\varepsilon }_{0}$$

4 $${\varepsilon {\prime\prime} }_{r}=4t/\pi \omega {R}_{P}{d}^{2}{\varepsilon }_{0}$$

### FTIR measurements

FTIR measurements performed on a Bruker Tensor 27 (Germany) FTIR spectrometer. To prepare samples for FTIR, 1 mg coal powder firstly mixed with potassium bromide (KBr) powder at a ratio of 1:100. The mixture then ground in an agate mortar for 6 min. Following grinding, the resulting powder mixture was placed in a mould and pressed into a round sheet under a pressure of 10 MPa. In the end, the scan of the sample performed in the wavenumber range of 400–4000 cm^−1^ to obtain absorption spectra with a resolution of 4 cm^−1^. The FTIR spectra were semi- quantitatively analyzed using the software OPUS (Bruker, Germany) and Omnic to get the component and structure change during early spontaneous combustion stage.

## Results and Discussion

### Dielectric properties of spontaneous combustion coal

**Figure 3:**
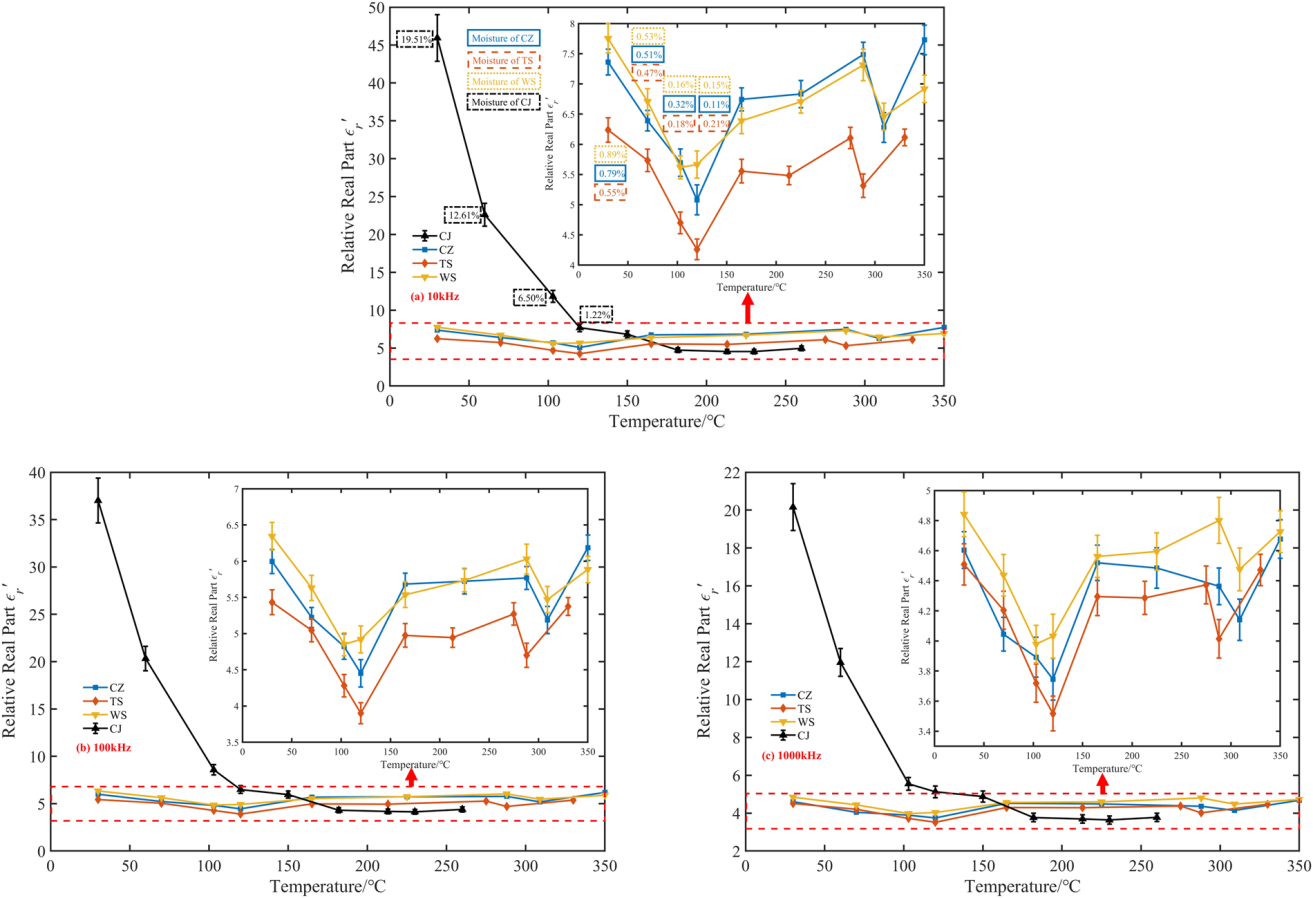
Temperature dependence of four coal dielectric constant real part at different frequencies. (**a**) 10 kHz, (**b**) 100 kHz, (**c**) 1000 kHz.

**Figure 4:**
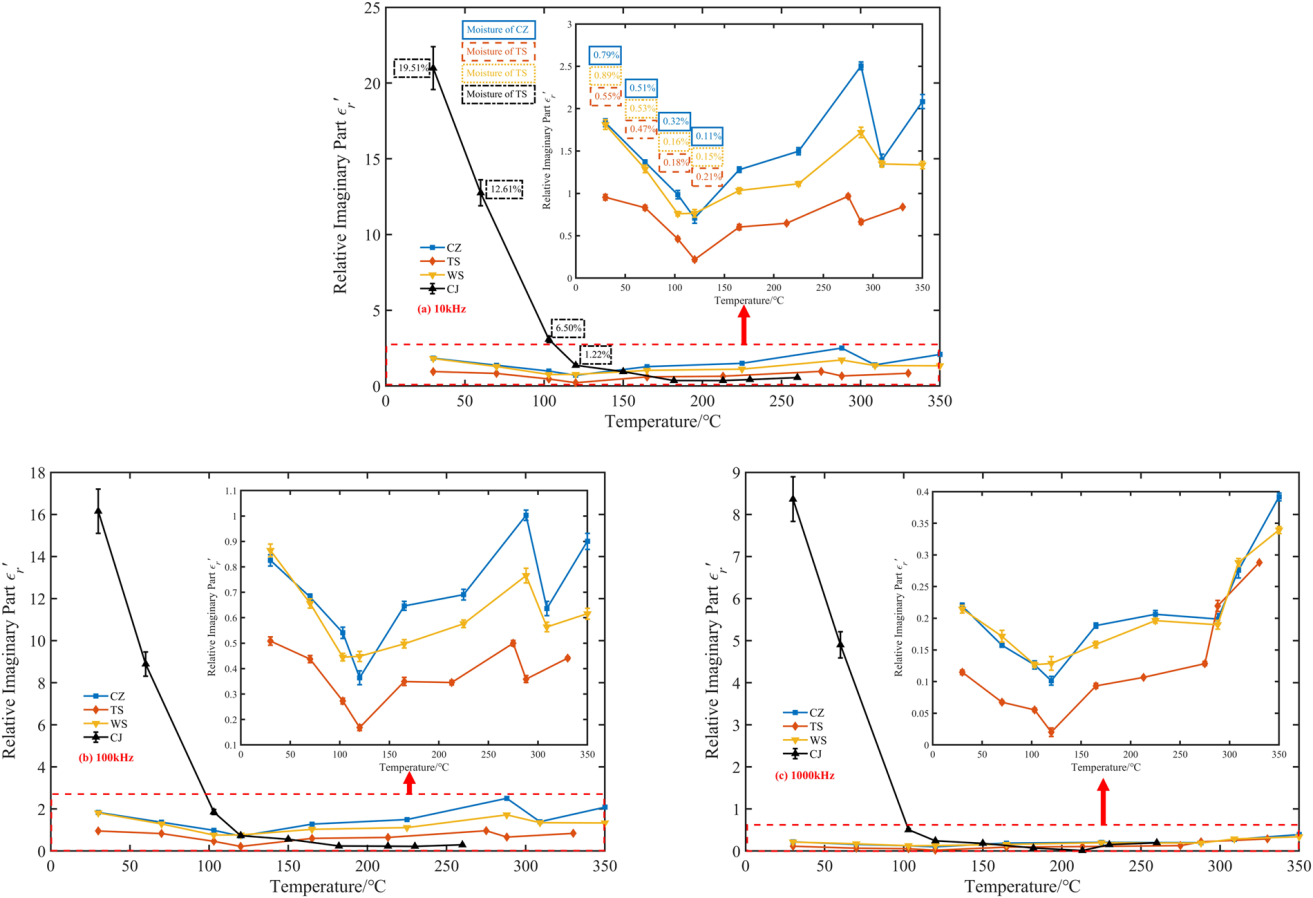
Temperature dependence of four coal dielectric constant imaginary part at different frequencies. (**a**) 10 kHz, (**b**) 100 kHz, (**c**) 1000 kHz.

The dielectric constant of spontaneous combustion coal at three intermediate frequency(10 kHz, 100 kHz, and 1000 kHz) was analyzed to reduce systematic errors. Figures 3 and 4 present the experimental data on the temperature dependence of real and imaginary dielectric constant of oxidized coal samples. Among the four raw coal samples, the dielectric constant of the CJ coal sample was the largest, about 45.94~20.16 in the test frequency range, and one order of magnitude higher than the real part of the dielectric constant of the other three coal samples. Followed by WS and CZ coal samples, their dielectric constant real parts were about 7.75 to 4.84 and 7.36 to 4.60, respectively. The real part of the dielectric constant of the TS coal sample was about 6.24 to 4.51, which was the smallest. The order of the imaginary and real dielectric constant values of four kinds of coal was the same. The imaginary part of the dielectric constant of CJ raw coal was about 20.98 to 8.36, WS raw coal was about 1.81 to 0.21, CZ raw coal was about 1.83 to 0.22, and TS raw coal was about 0.95 to 0.11. According to the proximate analysis results of the coals, the water contents of the CJ, WS, CZ, and TS raw coal were 19.51%, 0.89%, 0.79%, and 0.55% respectively, which was consistent with the coal relative dielectric constant order. A possible explanation for this might the fact that water is a highly polar material, which has a relative dielectric constant of approximately 80 at normal temperature and pressure. As the coal samples contained an amount of water at the initial temperature, especially CJ coal sample, they manifested high real and imaginary parts of the dielectric constant.

The change of the dielectric constant of the coal sample after heat treatment could divide into two stages. 30 °C to 120 °C corresponded to the coal dehydration stage, and 120 °C to *T*_*3*_ corresponded to the oxygen absorption stage. At the three frequencies, the real and imaginary parts of the dielectric constant of CJ coal samples dropped sharply by 83.3~74.6% and 93.5~97.1% respectively at 30 °C to 120 °C, then remain almost unchanged from 120 °C to *T*_*3*_. We could see that due to the high initial dielectric constant of the CJ coal sample, the variation characteristics of the remaining three coal samples with temperature were masked. Therefore, we separated the test results of WS coal, TS coal, and CZ coal samples. From Figs. 3 and 4 we can also see that the real and imaginary part of the dielectric constant of these three kinds of coal samples had a similar change trend with the rising temperature. The real part of the dielectric constant of WS coal, CZ coal, and TS coal sample decreased monotonously by 27.0~16.7%, 31.0~18.5% and 31.7~22.0% between 30 °C and 120 °C. The imaginary part of three coal samples decreased monotonously by 58.0~38.1%, 61.2~54.5% and 76.8~81.8%.

There are two possible explanations for the decrease of the complex relative dielectric constant with the increase of temperature from 30 °C to 120 °C. A possible reason for this may be the water evaporation. Another possible explanation for this is that water evaporates from pores. These pores were equivalent to a large number of small capacitors embedded in the coal. Because of these capacitors were air, which had a low dielectric constant, the overall relative dielectric constant of the coal decreased. However, for the volume of the pores were much smaller than the volume of the coal itself, the effect of the pore volume on the coal relative dielectric constant may not be significant. Besides, based on GF-A6 type industrial analyzer, the moisture content of coal samples before 120 °C was measured, as shown in Figs. 3(a) and 4(a). With the increase of temperature from 30 °C to 120 °C, the moisture content of CJ, WS, CZ and TS coal samples decreased by 18.29%, 0.74%, 0.68%, and 0.34% respectively. It could be seen that the higher the original moisture content of the coal sample, the more significant the decrease of the real and imaginary parts of the coal sample dielectric constant. The dielectric constant of the CJ coal sample decreased by more than 74.6% in the test frequency range^15^. Heated and then cooled a coal sample that contains a moisture content of 4%. He found that the dielectric constant of coal decreased from 2.69 to 2.35, a decrease of approximately 12.6%. Compared with his research results, the reduction of the dielectric constant of the coal sample in our experiments was 16.7% to 83.3%, which is obviously higher than 12.6%. The reason for this phenomenon was that the test frequency used in our research was lower than the 2.216 GHz he used. Our experimental results can also verify this phenomenon. For example, the CJ coal real dielectric constant was 47 at 10 kHz. It decreased to 37 at 100 kHz. However, the frequency had no discernible effect on the changing trend of coal dielectric constant during spontaneous combustion. In summary, the higher the moisture content in raw coal, the more likely it is to use electromagnetic method to predict the coal fire. At the same time, using a low-frequency electromagnetic wave as far as possible under the premise of ensuring the resolution may improve the accuracy of fire detection.

Coal rank and mineral composition may also important factors affecting the dielectric properties of coal. The dielectric constant of dry coal samples should be used as moisture has a very significant effect on the dielectric properties of coal, when discussing the effect of coal rank and ash on the dielectric constant of coal samples. From Figs. 1 and 3, one could see that the dehydration temperature of CJ coal samples is about 180 °C, and that of TS, CZ, and WS coal samples is about 120 °C. Take 10 kHz as an example, the real part of the dielectric constant of the dried WS coal sample is the largest, about 5.67, followed by CZ, CJ, and TS, and the real parts of their dielectric constants are 5.08, 4.73, and 4.26, respectively. The imaginary part of the dielectric constant of the dried WS coal sample is the largest, about 0.76, the next are CZ, CJ, and CJ, their imaginary parts of the dielectric constant are 0.71, 0.36, and 0.22. These results indicate that the higher the coal rank, the larger the real and imaginary dielectric constants. Because the higher the coal rank, the higher the degree of aromatization and more out-of-domain electrons could be generated when an electric field is applied. Mineral content in coal is mainly characterized by ash. According to the results of the industrial analysis in Table 1, the coal with the highest ash content is WS coal, which is about 13.97%, followed by CZ, TS, and CJ, which have ash contents of 10.84%, 10.00%, and 5.72%, respectively. The results show that the higher the ash content, the greater the dielectric constant of the dehydrated coal sample.

The dielectric constants of WS coal, CZ coal, and TS coal samples reached minimum values at 120 °C, which were 5.66–4.03, 5.08–3.75, and 4.26–3.52, respectively. The real and imaginary parts of the dielectric constant of each coal sample increased non-monotonically between 120 °C and T3, and the maximum value obtained at the highest three temperatures. However, the corresponding temperatures of the maximum values were different, which might be caused by systematic errors in measurement. The fluctuating growth in the coal relative dielectric constant after 120 °C could attribute to the chemical oxygen absorption reaction of coal. The active reductive groups reacted with oxygen during temperature increasing, and the carbonyl (C=O) compounds (such as aldehydes (RCHO), ketones (RCOR’), carboxylic acids (RCOOH), and carboxylic acid derivatives), ethers (C–O), and non-metallic oxides (Si–O) were generated^26,27^. These oxygen-containing compounds may have a relatively strong polarization response under the action of an electric field, which lead to an increase in the dielectric constant of oxidized coal samples.

As the coal oxidizes, the temperature of the coal rises, the water evaporates, and the coal becomes dry. The dried coal is further oxidized to become oxidized coal. The experimental results show that raw coal, dry coal, and oxidized coal have different dielectric constants, which meets the basic requirements of electromagnetic detection. Electromagnetic waves reflect at the interface between two materials with different dielectric constants, and the more significant the difference between the dielectric constants of the two materials, the more obvious the reflection is. If an area with potential coal spontaneous combustion risk continuously monitored and the amplitude and phase of the time-domain electromagnetic signals change, it may be that coal oxidation occurs in this area.

**Figure 5:**
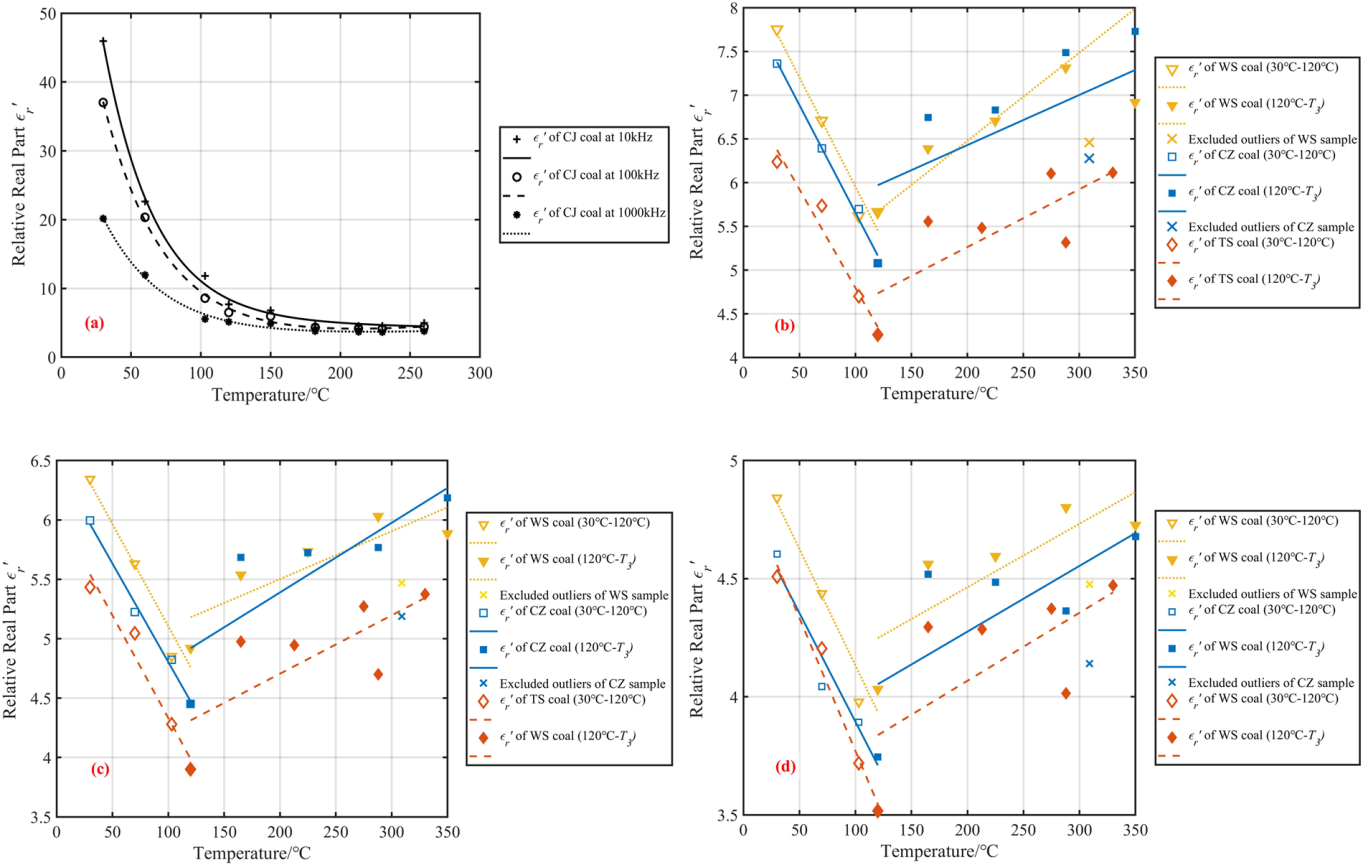
Fitting curves of the real part of the dielectric constant as a function of temperature. (**a**) The fitting curve of CJ coal sample at three frequencies, (**b**) Fitting curves of WS, CZ, and TS coal samples at 10 kHz, (**c**) Fitting curves of WS, CZ, and TS coal samples at 100 kHz, (**d**) Fitting curves of WS, CZ, and TS coal samples at 1000 kHz.

**Figure 6:**
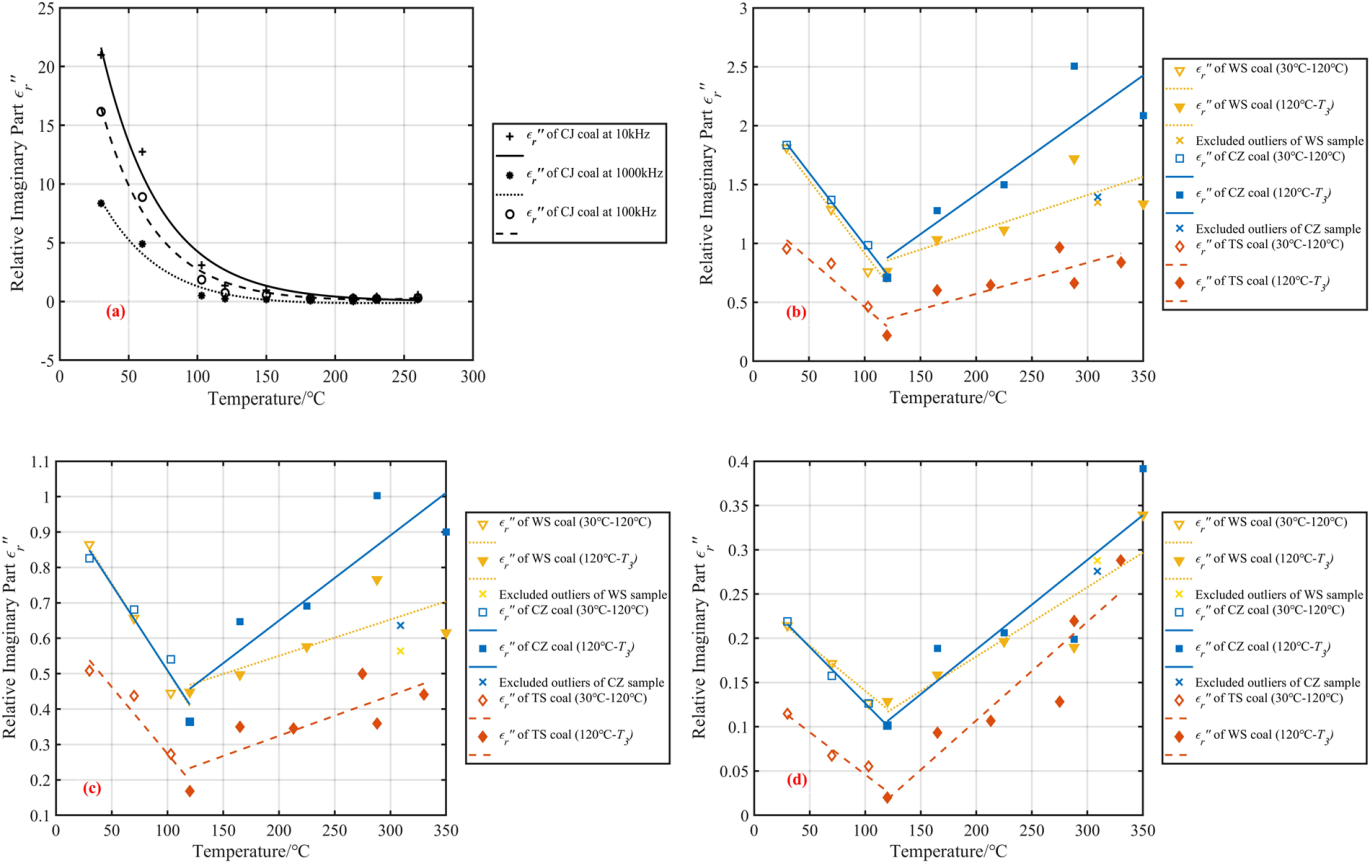
Fitting curves of the imaginary part of the dielectric constant as a function of temperature. (**a**) The fitting curve of CJ coal sample at three frequencies, (**b**) The fitting curve of WS, CZ, and TS coal samples at 10 kHz, (**c**) The fitting curve of WS, CZ, and TS coal samples at 100 kHz, (**d**) The fitting curve of WS, CZ, and TS coal samples at 1000 kHz.

In a previous work^28^, we proposed a model for the change of dielectric constant with temperature without considering moisture content. However, from Figs. 3 and 4, we found that high water content could conceal the change of dielectric constant with the increasing temperature. So it was necessary to discuss CJ coal samples with higher water content and WS, CZ and TS coal samples with lower water content, respectively. The simple statistical fitting analysis method was used to evaluate the relationship between dielectric constant and temperature further. Both the dielectric constant of CZ and WS coal samples became smaller abnormally at 320 °C. The data abnormity was considered to be caused by the preparation process for these two kinds of coal samples were produced at the same temperature, and therefore, these data were omitted. The results of the fitting analysis of the coal real and imaginary dielectric constant and temperature at different frequencies were summarised in Figs. 5 and 6. As can be seen from Figs. 5(a) and 6(a), the value of real and imaginary dielectric constants of CJ coal samples, with high water content, could be described by a binomial, exponential model during coal low-temperature oxidation process. The fitting equation is shown as Eq. (5), where $g(f)$ is a frequency correction function of coal dielectric constant. The correlation coefficient R^2^ and parameters are shown in Tables 3 and 4.

5 $${\varepsilon {\prime} }_{r}({\varepsilon {\prime\prime} }_{r})(f,T)=g(f)(a{e}^{bT}+c{e}^{dT})$$

**Table 3:** The correlation coefficient R^2^ and parameters of the fitting curves of the real part of dielectric constant.

Coals	30 ≤ *T* ≤ *T*_*3*_						
	f (kHz)	a	b	c	d	R^2^	
CJ	10	91.4	−0.02704	5.327	−0.0007381	0.9978	
	100	70.09	−0.02291	1.7	0.003538	0.9985	
	1000	35.2	−0.02311	2.543	0.001438	0.996	
		**30 ≤ *T* ≤ 120**			**120 ≤ *T* ≤ *T***_***3***_		
		***p***	***q***	**R**^**2**^	***r***	***s***	**R**^**2**^
WS	10	−0.02501	8.455	0.9625	0.00573	5.282	0.7282
	100	−0.01722	6.827	0.9544	0.00403	4.696	0.7395
	1000	−0.009869	5.119	0.9479	0.002689	3.926	0.6767
TS	10	−0.02249	7.049	0.9618	0.006601	3.942	0.6032
	100	−0.01726	6.059	0.9609	0.004914	3.723	0.5522
	1000	−0.01125	4.896	0.9791	0.002878	3.492	0.4338
CZ	10	−0.02457	8.116	0.9928	0.01008	4.461	0.8072
	100	−0.01657	6.462	0.9908	0.005864	4.216	0.6886
	1000	−0.00921	4.815	0.9475	0.002782	3.719	0.5083

**Table 4:** The correlation coefficient R^2^ and parameters of the fitting curves of the imaginary part of dielectric constant.

Coals	30 ≤ *T* ≤ *T*_*3*_						
	f (kHz)	a	b	c	d	R^2^	
CJ	10	43.37	−0.02321	0	0	0.983	
	100	35.64	−0.02564	2.617e-06	0.04429	0.9879	
	1000	9.486e + 04	−0.01686	−9.484e + 04	−0.01686	0.9771	
		**30 ≤ *T* ≤ 120**			**120 ≤ *T* ≤ *T***_***3***_		
		***p***	***q***	**R**^**2**^	***r***	***s***	**R**^**2**^
WS	10	−0.01246	2.163	0.9683	0.003096	0.4825	0.6371
	100	−0.004963	1.004	0.9672	0.001025	0.3451	0.5967
	1000	−0.001027	0.2432	0.9623	0.0007824	0.0229	0.7938
TS	10	−0.008145	1.274	0.9161	0.002646	0.04267	0.6887
	100	−0.003776	0.6515	0.939	0.001137	0.09678	0.6544
	1000	−0.0009583	0.1418	0.9458	0.001115	−0.1158	0.8642
CZ	10	−0.01229	2.217	0.9963	0.006745	0.06616	0.7913
	100	−0.004845	0.9941	0.9513	0.002407	0.1682	0.8052
	1000	−0.001274	0.2539	0.9901	0.001012	−0.0151	0.7744

It was clear that there was an excellent agreement between the experimental data and binomial, exponential regression equation with a correlation coefficient higher than 99%. The preexponential factor *a* was observed to be related to the dielectric constant at room temperature. The larger the initial dielectric constant, the larger the value of *a*, and vice versa. Therefore, it could be concluded that the value of *a* depended on the moisture content of coal samples and *b* depended on the loss rate of water. The second term of the regression equation mainly played a role in correcting the high-temperature section.

Figures 5(b–d) and 6(b–d) show the fitting curve of the real and imaginary part of WS, CZ, and TS coal dielectric constant with the temperature respectively. Due to the low water content of these three coal samples, the coal dielectric properties between 120 °C and T3 could not be ignored. Curve fitting results showed that a piecewise linear model could describe the real and imaginary parts of the dielectric constant. The linear regression equation is shown as Eq. (6), where $g(f)$ is a frequency correction function of coal dielectric constant.and the correlation coefficient R^2^ and parameters are shown in Tables 3 and 4.

6 $${\varepsilon {\prime} }_{r}({\varepsilon {\prime\prime} }_{r})(f,T)=\{\begin{array}{c}g(f)(pT+q)\,(30\,^\circ {\rm{C}}\le T\le 120\,^\circ {\rm{C}})\\ g(f)(rT+s)\,(120\,^\circ {\rm{C}}\le T\le {T}_{3})\end{array}$$

As Tables 3 and 4 show, there was a significant negative correlation between temperature and coal dielectric constant between 30 °C and 120 °C for the correlation coefficient of the linear regression equation was more than 94%. It was likely that the slope of the linear regression equation *p* and the intercept *q* was determined by the coal water content and the waterfree coal dielectric constant. For example, TS coal sample had the lowest initial dielectric constant, that was to say, the coal sample had the lowest moisture content. However, the slope of the fitting equation was almost the same as WS and CZ coal sample. The reason for this was that the waterfree dielectric constant of TS coal sample was the lowest. The correlation coefficients of the regression equations between 120 °C to *T*_*3*_, shown in Tables 3 and 4, were higher than 60% except for the TS coal sample at 100 kHz and 1000 kHz. It seemed that a strong positive linear relationship existed between the coal dielectric properties and temperature during the oxygen adsorption stage (120 °C to *T*_*3*_) although it was not as apparent as the dehydration stage. The slope *r* and intercept *S* of the linear regression equation of the low-temperature oxidation stage may depend on the oxidation characteristics of coal (e.g., different reactions in the low-temperature oxidation process, types, and contents of oxygenated compounds).

### Changes in the functional groups of oxidized coal

The type and content of functional groups, which is a critical characterization of the coal composition and structure, vary with different stages of coal oxidation. Changes in the coal component and construction can cause changes in the relative dielectric constant. Consequently, it is necessary to assess the evolution of coal functional groups during low-temperature oxidation to comprehend the causes of changes in dielectric properties. Many scholars have used FTIR spectrometry to characterize the component and structure of coal. This technique is also employed in this study.

**Figure 7:**
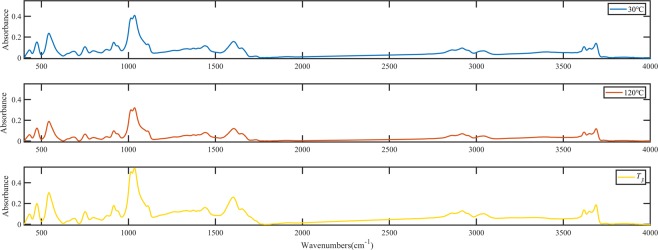
FTIR spectra of oxidized WS coal samples at 30 °C, 120 °C, and *T*_*3*_.

The infrared spectra of the WS, CZ, and TS coal samples at the start and stop temperatures of the water loss and oxygen absorption stages were measured. Take the WS coal as an example. The original FTIR spectra of WS coal sample treated under 30 °C, 120 °C, and *T*_*3*_ are shown in Fig. 7. Each FTIR spectrum for the oxidized coal samples in Fig. 7 has two main oxygen-containing functional groups regions: the ether region (from 1300–1110 cm^−1^) and the carbonyl region (from 1650–1750 cm^−1^)^29–32^.

**Figure 8:**
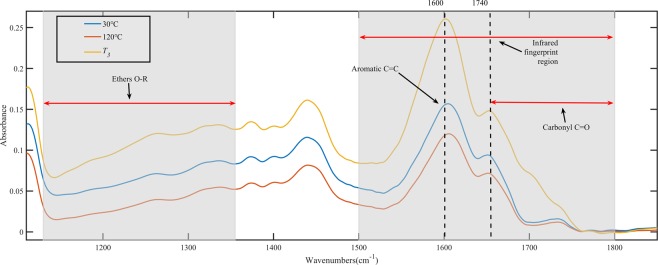
FTIR spectra of main oxygen-containing functional groups of oxidized WS coal samples at test temperatures.

**Figure 9:**
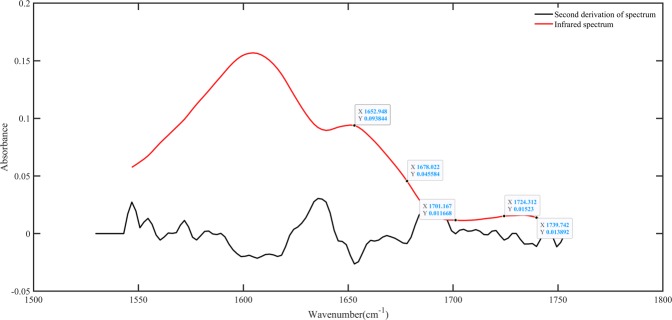
Curve-resolved spectra for WS raw coal in the region of 1800–1600 cm^−1^.

**Figure 10:**
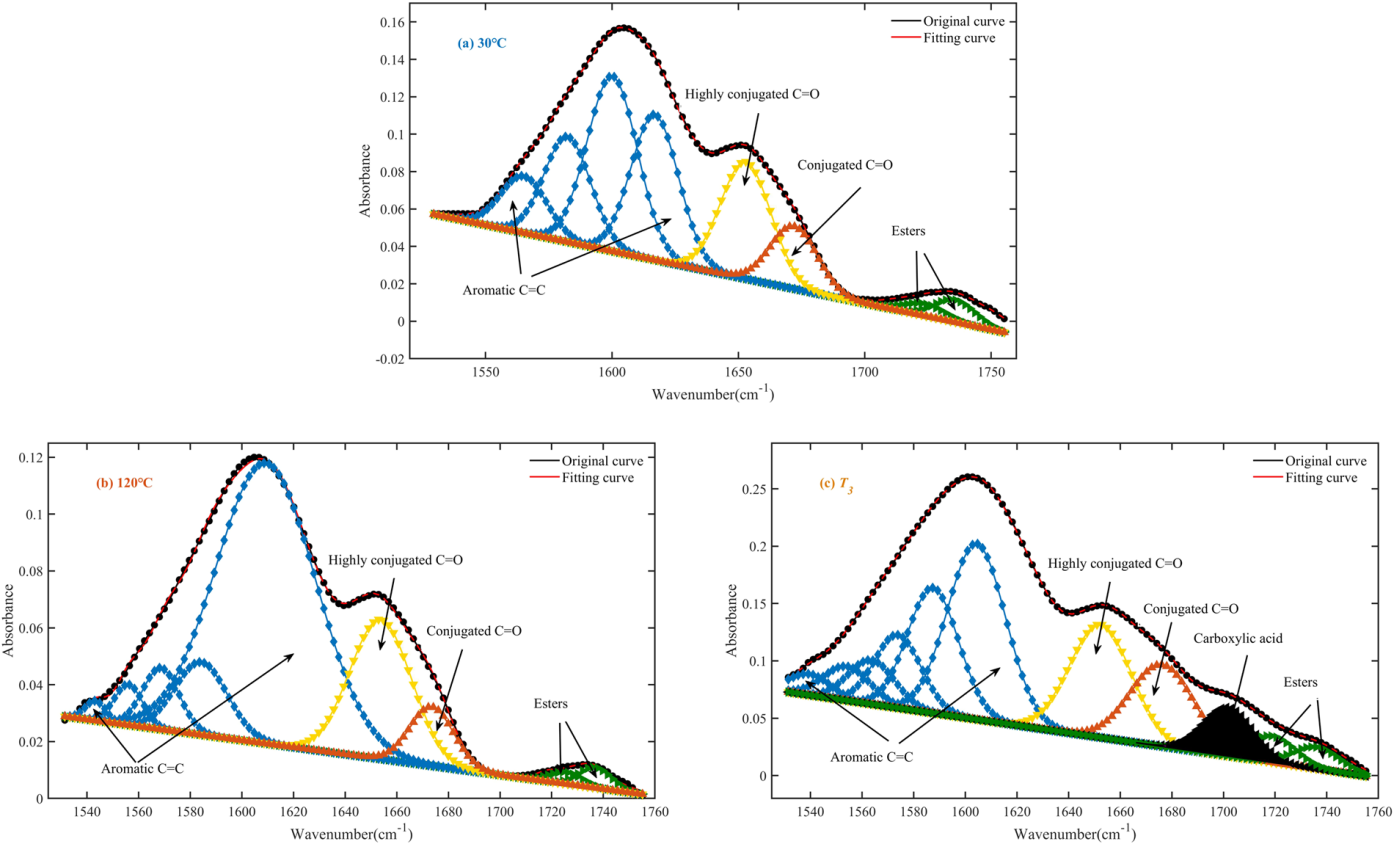
Curve-fitted spectra in the region of 1800–1600 cm^−1^ for 3 temperatures. (**a**) 30 °C, (**b**) 120, and (**c**) *T*_*3*_.

The FTIR spectra of the main oxygen-containing functional groups are shown in Fig. 8. From the graph, we can see that the bandwidth and absorbance of the oxygen-containing functional groups in the coal sample increased with increasing temperature. The peaks were deconvoluted and fitted to compare the difference in oxygen-containing functional groups among various coal samples in more detail^33–35^. Take WS coal sample as an example; the deconvolution results of carbonyl functional groups region are shown in Fig. 9. The results revealed 4 to 5 absorption peaks in the area of 1800–1600 cm^−1^, which were attributed to different carbonyl (C=O) compounds. Gaussian-type curves were used for all the peaks, and the baseline was defined as linear. The parameters used for the peak fitting are shown in Table 5. The difference in the transparency of the coal sample caused the baseline of the infrared spectrum to tilt, resulting in a slight shift in the center of the fitted peak of the functional group in different coal samples. The curve-fitted spectrum of WS coal in the region of 1800^−1^ to1600^−1^ is shown in Fig. 10. As shown in Fig. 10, with increasing temperature, the carboxylic acid (RCOOH) groups increased most significantly among all carbonyl compounds. From 30 °C to 120 °C, the carboxylic acid group of WS hardly showed in the FTIR spectra. However, with oxidation temperature exceeded 120 °C, the absorption peak area for the carboxylic acid group (1701 cm^−1^) increased obviously, indicating that the carboxylic acid group content increases significantly. As can be seen in Fig. 8, the peak area of ethers vary slightly between 30 and 120 °C. When the temperature reaches *T*_*3*_, the peak value and peak area increased significantly compared with 120 °C. The peaks in the ethers region (between 1300 and 1100 cm^−1^) separated clearly, and thus need not to carry out peak fitting.

**Table 5:** Peak fitting parameters for the FTIR spectrum of WS raw coal in the region of 1800–1600 cm^−1^.

Peak	Center (cm^−1^)	Assignment
1	1740	Esters
2	1724	Esters
3	1701	Carboxylic acid
4	1678	Conjugated C=O
5	1653	Highly conjugated C=O

A semi-quantitative calculation was made based on the absorbance curves to investigate changes in the relative content of oxygen-containing groups. The C=C functional group was considered to be constant during coal low-temperature spontaneous combustion^36–38^. Thereby, the peak area ratio of the oxygen-containing functional groups and C=C groups can adequately characterize the relative content of the oxygen-containing functional group during the oxidation process. The following relationships were investigated:

(C=O + O − R)/C=C((1650–1750 cm^−1^) band + (1300–1110 cm^−1^) band)/(1550–1605 cm^−1^) band

**Figure 11:**
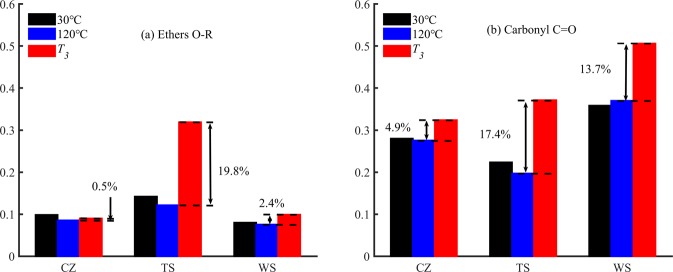
The relative content of coal oxygen-containing functional groups. (**a**) Ethers; (**b**) Carbonyl compound.

Figure 11 shows the relative content of ethers and carbonyl compounds. As shown in Fig. 11, the relative content of ethers in coal was lower than that of carbonyl compounds. The relative content of both ethers and carbonyl compounds of coal samples at 30 °C and 120 °C did not change remarkably. We can also see from the figure that with the oxidation temperature reached to *T*_*3*_, the relative content of ethers in CZ, TS, and WS coal samples increased by 0.47%, 19.7%, and 2.37%, respectively; except for TS coal samples, the relative content of CZ and WS increased slightly. The relative content of carbonyl compounds in three coal samples increased by 4.88%, 17.41%, and 13.71%, respectively, and TS coal sample increased the most.

### Correlation between structural and material changes and dielectric response to coal low temperature oxidation

**Figure 12:**
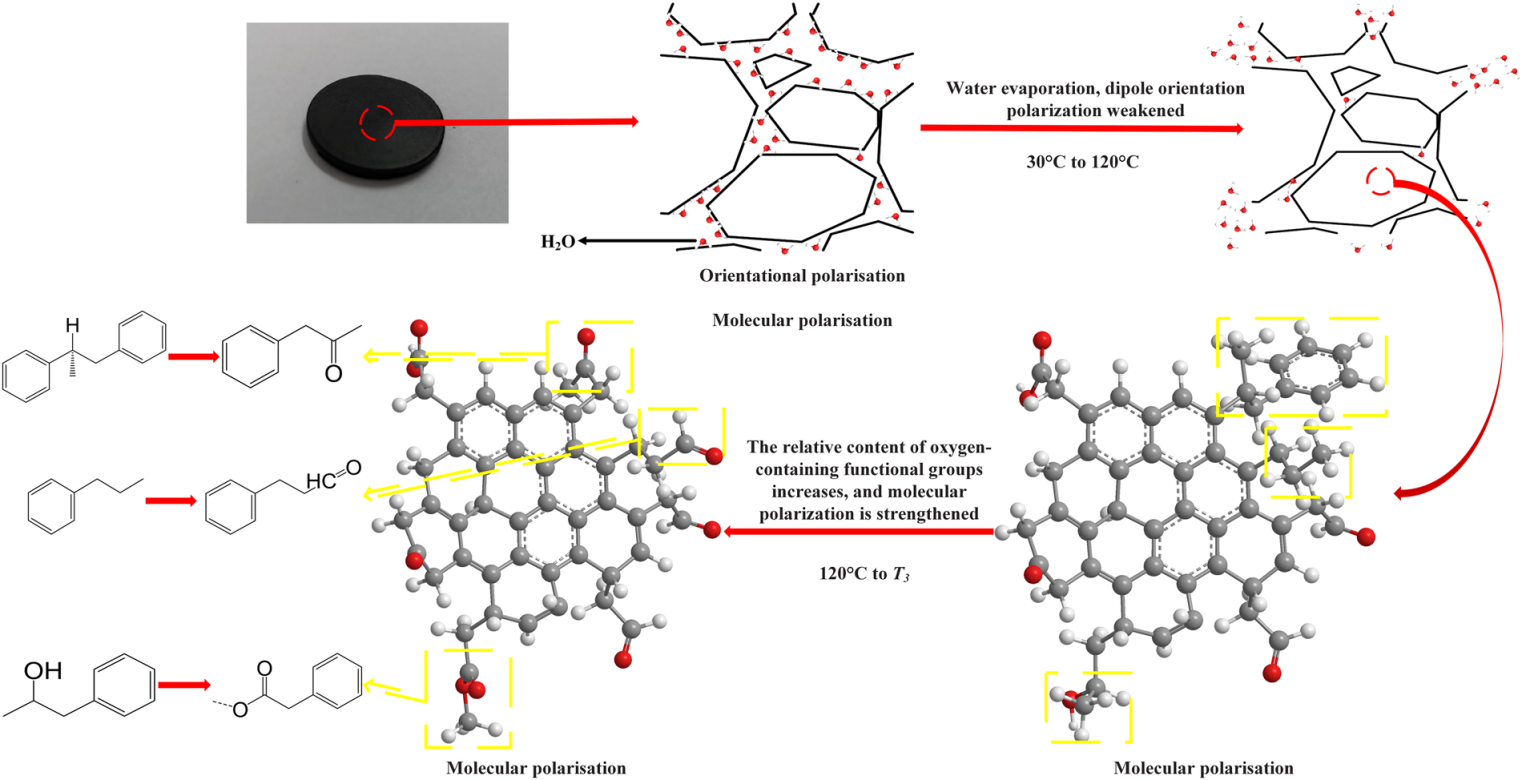
Schematic diagram of coal material and structure evolution during low-temperature oxidation.

A schematic diagram of the changes in structure and product during spontaneous combustion of coal was plotted to illustrate the effect on dielectric properties, as shown in Fig. 12.

The water molecule in raw coal produces intense dipole orientation polarization under the action of the external electric field, so the dielectric constant of raw coal is the largest. Besides, coal is a cross-linked macromolecule that contains only a small amount of dipoles. The dipole has little freedom of movement, which leads to weak orientation polarization. Therefore, after the raw coal loses water, the coal dielectric constant reaches a minimum. Carbon-carbon $\pi$-bonds exist between aromatic carbon layers, and the electron of the $\pi$-bond in crystalline carbon was a type of localized electron^39–41^. Localized electrons remain in localized sites most of the time, but can occasionally jump to neighboring localized sites, which could lead to polarization under certain boundary conditions. The probability of these jumps occurring can be determined based on the distance between the two locations. Some studies proved that the aromatic structure of coal becomes denser after high-temperature treatment. It makes the jump of electrons more likely to occur, and thus increasing the dielectric constant of coal. However, for low-temperature spontaneous combustion coal, the aromatic structure in coal changes little. As a result, the effect of oxidation on coal dielectric constant could be explained from the oxidation product. With the oxidation of coal, the hydrogen peroxide radical generated from the aliphatic side chains combines with a hydrogen atom to form a peroxide (–O–O–) and a new carbon atom center. The hydrogen peroxide then formed hydroxyl (–OH) and ethers (C–O). Finally, the hydroxyl (–OH) decomposed and regenerated into the carbonyl group. The polarization of material can be calculated as the sum of the orientation polarization and the induced dipole moment^42^:

11 $$\chi ={N}_{d}{\mu }^{2}/3{\varepsilon }_{0}kT+{N}_{m}\alpha $$

In Eq. (11), ${N}_{d}$ and ${N}_{m}$ are the numbers of permanent dipoles and polarizable molecules, respectively. *T* is the temperature, $\mu$ is the dipole moment, *k* is the Boltzmann constant, and $\alpha$ is the polarizability of the molecule. Average values of typical bond polarizabilities and typical values for the permanent dipole moments of bonds and molecules are shown in Table 6. ^42^. In Table 6, ${\alpha }_{M}$ is the mean polarizability for all three directions, in units of A^3^.

**Table 6:** Polarizabilities and dipole moments of chemical bonds.

Chemical bonds polarizabilities			Chemical bonds dipole moments	
Bond	${{\boldsymbol{\alpha }}}_{{\boldsymbol{M}}}$	Comments	Bond	*μ*.Debye (D)
C–H	0.65	aliphatic	C–H	0.4
C–C	0.64	aliphatic	O–H	1.51
C–C	1.07	aromatic	C–C	0
C=C	1.66		C–O	0.74
C=O	1.20	carbonyl	C=O	2.3

As can be seen from Table 6, the mean polarizability of carboxyl groups (C=O) is 1.20 A^3^. It is almost twice the average value of the aliphatic carbon-hydrogen bond (C–H). We could also see that the dipole moment of carboxyl (C=O) was 2.3 Debye, which is about six times the dipole moment of the carbon-hydrogen bond (C–H) and three times of the ethers bond. As a result, both the orientation polarization and the induced dipole moment of oxidized coal samples increased significantly because of the increase of the oxygenated compounds. Thus the polarization and dielectric response of coal samples determined by the superposition of both effects were enhanced. We can also suggest that carbonyl compounds play the most significant role in dielectric response of coal among the oxygenated compounds. Because in this study, the relative content of carbonyl compounds had been found higher than that of ethers. Though surprisingly the observed relative content of ethers in TS coal increased significantly at *T*_*3*_ temperature, the increase of carbonyl compounds was generally more significant than that of ethers. And in section 3.1, no significant increase in the slope of the linear regression equation was observed along with the considerable increase of relative content of ethers of TS coal during 120 °C to *T*_*3*_. It further proves that the effect of ethers on the dielectric constant of self-ignited coal is less significant than that of carbonyl compounds.

It can also be seen from Eq. (11) that the self-ignited coal dielectric property was closely related to the real-time temperature. The coal dielectric properties were mainly studied from the oxidation product point of view because of the limitation of current experimental instruments. As the water evaporates during spontaneous coal combustion, the orientation polarization of the coal sample weakens. While the induced polarization is relatively less affected by temperature. As a result, even if the temperature was not considered, the results still could reflect the variation of dielectric properties of coal with temperature. Further research should be done to investigate the real-time dielectric properties changes during coal spontaneous combustion process.

## Conclusion

This study had established a temperature-dependent dielectric constant model of spontaneous combustion coal based on different moisture content and explained the principles of these changes from oxidation products. The following main conclusions were drawn:

1. Based on different water contents, the relationship between coal dielectric constant and temperature during spontaneous combustion was described by different mathematical models. There was a significant binomial, exponential relationship between dielectric constant and temperature (30 °C to *T*_*3*_) for high water CJ coal, the moisture of which is 19.51%. For CZ, WS and TS coal with lower water content, the moisture of which range from 0.89% to 0.55%, there was an apparent piecewise linear relationship between dielectric constant and temperature. The coal dielectric constant was negatively linearly correlated with the temperature at the low-temperature stage (30 °C to120 °C) and positively linearly correlated with the temperature at the high-temperature (120 °C to *T*_*3*_), respectively.
2. For CJ coal samples with 19.51% water content, the dielectric constant decreased by 74.6% due to temperature rise and water loss. While for the other three coal samples with about 1% water content, the dielectric constant decreased by only 16.7% to 31.7%. Besides, reducing the detection frequency under the premise of the resolution is beneficial to amplifying the water loss effect of coal. Therefore, we believe that using low-frequency electromagnetic waves to predict coal fire with higher water content can achieve better results.
3. Dehydration was the reason for the decrease of coal dielectric constant in the low-temperature stage. The increase of the relative content of oxygen-containing functional groups, especially carbonyl compounds, was the reason for the rise in the coal dielectric constant in the high-temperature stage. Therefore, for coals with lower water content, the increase of the dielectric constant during low-temperature oxidation(120 °C to *T*_*3*_) can be used as a supplement to enhance the reliability of prediction.

## Data Availability

The datasets generated and/or analyzed during the current study are available from the corresponding author on reasonable request.

## References

[1] Song Z, Kuenzer C (2014). Coal fires in China over the last decade: A comprehensive review. International Journal of Coal Geology.

[2] Kuenzer C, Stracher GB (2012). Geomorphology of coal seam fires. Geomorphology.

[3] Xin, M. *Segmented Electrical Characteristic of Coal Spontaneous Combustion and Transient Electromagnetic Detection Technology on Localization of Combustion Area in Coal Mine*, China University of Mining and Technology (Beijing) (2016).

[4] Yang F, Peng SP, Jian-Wei MA, Shuang HE (2010). Spectral analysis for ground penetrating radar surveys of the underground coal fire in Wuda Coal Mine. Journal of China Coal Society.

[5] Gundelach, V. In *Latest Developments in Coal Fire Research—Bridging the Science, Economics, and Politics of a Global Disaster* pp. 93–98 (Berlin, 2010).

[6] Catapano I, Affinito A, Moro AD, Alli G, Soldovieri F (2015). Forward-Looking Ground-Penetrating Radar via a Linear Inverse Scattering Approach. IEEE Transactions on Geoscience & Remote Sensing.

[7] Epov MI, Morozova GM, Antonov EY, Kuzin IG (2003). Method of Nondestructive Testing for Technical State of Casing Strings in Oil-and-Gas Wells Basing on the Transient Electromagnetic Method. Journal of Mining Science.

[8] Han D, Dan L, Shi X (2011). Effect of Application of Transient Electromagnetic Method in Detection of Water-Inrushing Structures in Coal Mines. Procedia Earth & Planetary Science.

[9] Hyun SY, Jo YS, Oh HC, Kim SY, Kim YS (2007). The laboratory scaled-down model of a ground-penetrating radar for leak detection of water pipes. Measurement Science & Technology.

[10] Trees, H. L. V. Ground Penetrating Radar Theory and Applications. **51**, 595–604 (2009).

[11] Peng Z, Hwang JY, Kim BG, Mouris J, Hutcheon R (2012). Microwave Absorption Capability of High Volatile Bituminous Coal during Pyrolysis. Energy & Fuels.

[12] Wang QD, Wang GH, Chen B, Wang SJ (2016). Permittivity‐Based Microwave Absorption Characteristics of Dongsheng Lignite during Pyrolysis. Energy Technology.

[13] Xu L (2014). Structural order and dielectric properties of coal chars. Fuel.

[14] Liang, X. U., Liu, H., Jin, Y., Fan, B. & Qiao, X. Experimental study on dielectric properties of coal chars under different char-making conditions. *Thermal Power Generation* (2015).

[15] Marland S, Merchant A, Rowson N (2001). Dielectric properties of coal. Fuel.

[16] Xu, H. Measurement and test of seam electric parameter and study on relationship between seam electric parameter and coal petrology characteristics. *Coal Science & Technology* (2005).

[17] Song Z, Fan H, Jiang J, Li C (2017). Insight into effects of pore diffusion on smoldering kinetics of coal using a 4-step chemical reaction model. Journal of Loss Prevention in the Process Industries.

[18] Song Z, Huang X, Luo M, Gong J, Pan X (2017). Experimental study on the diffusion–kinetics interaction in heterogeneous reaction of coal. Journal of Thermal Analysis and Calorimetry.

[19] Song Z, Wu D, Jiang J, Pan X (2019). Thermo-solutal buoyancy driven air flow through thermally decomposed thin porous media in a U-shaped channel: Towards understanding persistent underground coal fires. Applied Thermal Engineering.

[20] Song Z, Huang X, Jiang J, Pan X (2020). A laboratory approach to CO_2_ and CO emission factors from underground coal fires. International Journal of Coal Geology.

[21] Chen Y, Mori S, Pan WP (1996). Studying the mechanism of ignition of coal particles by TG-DTA. Thermochimica Acta.

[22] Marinov SP (2010). Combustion behaviour of some biodesulphurized coals assessed by TGA/DTA. Thermochimica Acta.

[23] Qu, L. *The study on the characteristics of coal stage and the critical point variation of the spontaneous combustion*, China University of Mining and Technology (Beijing) (2013).

[24] Yu, M. *Prevention and Control of Mine Fire*. 213 (National Defence Industry Press, 2013).

[25] Song, Z. *et al*. Chimney effect induced by smoldering fire in a U-shaped porous channel: A governing mechanism of the persistent underground coal fires. *Process Safety and Environmental Protection* (2020).

[26] Zhang WQ (2011). B-Mode Grey Relational Analysis of Surface Functional Groups Change Rules in Coal Spontaneous Combustion. Advanced Materials Research.

[27] Zhou C (2017). Study on the relationship between microscopic functional group and coal mass changes during low-temperature oxidation of coal. International Journal of Coal Geology.

[28] Zhu H, Wang W, Wang H, Zhao H, Xin M (2018). Study on electrical properties of coal at spontaneous combustion characteristic temperature. Journal of Applied Geophysics.

[29] Geng W, Nakajima T, Takanashi H, Ohki A (2009). Analysis of carboxyl group in coal and coal aromaticity by Fourier transform infrared (FT-IR) spectrometry. Fuel.

[30] Qi X, Wang D, Xin H, Qi G (2014). An *In Situ* Testing Method for Analyzing the Changes of Active Groups in Coal Oxidation at Low Temperatures. Spectroscopy Letters.

[31] Xu T (2014). *In-situ* Series Diffuse Reflection FTIR Used in Studying the Oxidation Process of Coal. Energy Sources.

[32] Zhang W (2015). Thermogravimetric Dynamics and FTIR Analysis on Oxidation Properties of Low-Rank Coal at Low and Moderate Temperatures. Coal Preparation.

[33] Ibarra J, Munoz V (1996). & Moliner. FTIR study of the evolution of coal structure during the coalification process. Organic Geochemistry.

[34] Arash T, Yu J, Han Y, Yin F, Stokie D (2012). Study of Chemical Structure Changes of Chinese Lignite upon Drying in Superheated Steam, Microwave and Hot Air. Energy & Fuels.

[35] Zhang Y (2016). Kinetic study on changes in methyl and methylene groups during low-temperature oxidation of coal via *in-situ* FTIR. International Journal of Coal Geology.

[36] Xu-Yao, Q. I. Oxidation and self-reaction of active groups in coal. *Journal of China Coal Society* (2011).

[37] Han F, Zhang YG, Meng AH, Qing-Hai LI (2014). FTIR analysis of Yunnan Lignite. Journal of China Coal Society.

[38] Lu X, Zhao H, Zhu H, Han Y, Xue X (2018). Characteristic rule of spontaneous combustion tendency of oxidized coal at recrudescence stage. Journal of China Coal Society.

[39] Cheng DK (1989). & Education, P. Field and Wave Electromagnetics:International Edition. Human Resource Development Quarterly.

[40] Forniésmarquina JM, Martín JC, Martínez JP, Miranda JL, Romero C (2003). Dielectric characterization of coals. Canadian Journal of Physics.

[41] Selim MM, El-Nabarawy TA, Ghazy TM, Farid T (1981). The relation between the adsorption characteristics of polar organic compounds (alcohols and acids) and their orientation polarization on activated carbon. Carbon.

[42] Jonscher, A. K. *Dielectric relaxation in solids*. 27–29 (2008).

